# Dissolving microdroplet electroanalysis enables attomolar-level detection

**DOI:** 10.1039/d5an00795j

**Published:** 2025-08-19

**Authors:** James H. Nguyen, Ashutosh Rana, Savannah M. Hatch, Jeffrey E. Dick

**Affiliations:** a Department of Chemistry, Purdue University West Lafayette IN 47907 USA jdick@purdue.edu; b Elmore Family School of Electrical and Computer Engineering, Purdue University West Lafayette IN 47907 USA

## Abstract

Trace detection is critical for identifying chemicals that would otherwise remain undetectable. While analytical techniques, such as spectroscopy, spectrometry, and electrochemical sensors, are effective at detecting low concentrations, achieving attomolar sensitivity remains a significant challenge. Here, we present an electroanalytical approach that leverages partitioning kinetics to detect attomolar concentrations of redox-active analytes. Using (Cp*)_2_Fe^II^ as a model system, we demonstrate trace-level detection by facilitating the transfer of (Cp*)_2_Fe^II^ from the bulk aqueous phase into 1,2-dichloroethane (DCE) microdroplets positioned atop a gold microelectrode (radius ∼6.25 μm). This partitioning arises from the greater solubility of (Cp*)_2_Fe^II^ in DCE relative to its limited solubility in water, enriching the analyte concentration near the electrode as the microdroplets slowly dissolve into the aqueous phase. Additionally, we explored the role of oxygen in enhancing the electrochemical response: oxygen removal hindered detection at 1 aM, while oxygen saturation significantly amplified the redox peak signal. These findings underscore oxygen's role, which is likely a bimolecular reaction between oxygen and (Cp*)_2_Fe^II^ in signal amplification. An EC’ catalytic mechanism likely amplifies the electrochemical signal of (Cp*)_2_Fe^II^ when the droplet is sufficiently small for feedback to occur, enabling attomolar detection of (Cp*)_2_Fe^II^. This study introduces a partitioning-based electroanalytical strategy taking advantage of an an EC’ catalytic mechanism for ultra-low detection limits, offering promising applications in trace chemical analysis and advanced sensor technologies.

## Abstract 2

We present an electrochemical platform for achieving attomolar detection by leveraging partitioning kinetics. The partitioning arises from the higher solubility of Cp_2_*(Fe)^II^ in DCE *versus* water, allowing enrichment of analyte concentration.

## Graphical abstract

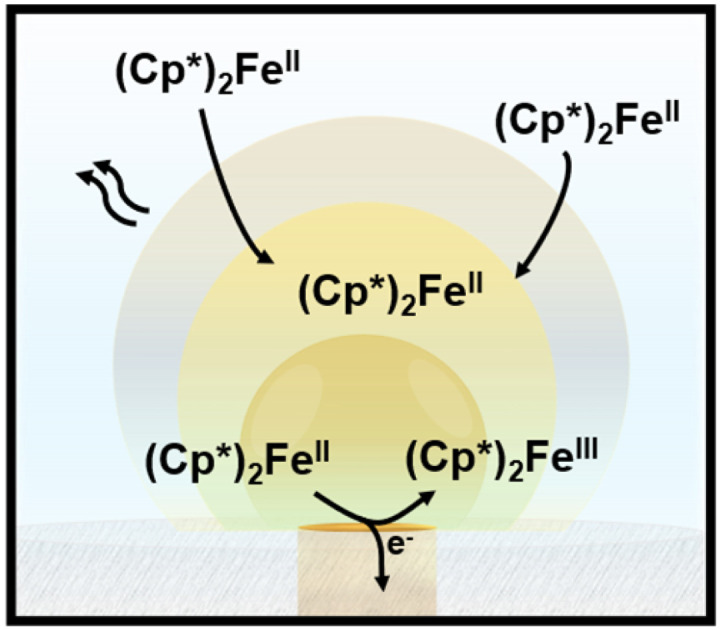

## Introduction

The detection of trace chemicals is crucial across various fields such as environmental monitoring, pharmaceuticals, food safety, and forensic science.^1,2^ This is important because it enables the detection, identification, and quantification of trace concentrations of chemicals and contaminants that might otherwise go undetected. Trace detection is essential for ensuring regulatory compliance, minimizing human exposure to toxins, and protecting ecosystems from pollutants. Several techniques relying on principles of spectroscopy and spectrometry in the field of analytical chemistry are widely utilized in trace detection due to their accuracy and reliability in identifying minute quantities of substances in complex mixtures.^3^

The most common spectrometric technique is inductively coupled plasma mass spectrometry (ICP-MS), which stands out for its exceptional sensitivity, with a low detection limit of ∼1 nmol L^−1^ of analyte.^4,5^ This allows detection of trace elements even at parts-per-trillion levels. This is critical in applications where even trace amounts can have significant biological or environmental impacts. ICP-MS maintains high precision in its measurements, ensuring that even at low concentrations, the results are reliable and reproducible. Other spectrometric techniques, such as gas or liquid chromatography coupled with mass spectrometry (GC-MS/LC-MS), excel at detecting compounds at extremely low concentrations (ppb to ppt).^5–7^ Additionally, spectroscopic techniques like atomic absorption spectroscopy (ppm to ppb) and fluorescence spectroscopy (ppm to ppb) can be valuable tools in the field of trace detection.^8–10^ Finally, a uniquely powerful method for trace element detection is droplet-based sensing by leveraging evaporation. This technique has even lower LOD levels when used in conjunction with surface-enhanced Raman spectroscopy (SERS).^11–13^ This approach takes advantage of distinctive properties of microdroplets like high droplet dissolution rates, allowing measurement of analyte concentration.^14–18^

The chemistry of microdroplets is fascinating because reactions within these tiny reactors often exhibit interesting behaviors that bulk solutions do not have. In microdroplets, there are unique phenomena that allow for novel discoveries such as reaction acceleration, unique transformation that is normally inaccessible, and enhanced sensing capabilities.^19–21^ Microdroplets have been shown to accelerate reactions by one or more orders of magnitude and certain reactions that normally do not occur in bulk can occur in microdroplets.^22–24^ They also have high sensing capabilities due to the microdroplet's high surface-area-to-volume ratio and their confined environment allows for low detection.^25,26^

Our group recently developed an innovative electrochemical platform for detecting trace analytes through droplet-based sensing that takes advantage of droplet dissolution in biphasic systems to concentrate analytes in a microdroplet.^27–31^ We were able to detect sub-nM levels of redox activity analyte and pushed the LOD levels to <1000 molecules of analyte in subsequent work using EC’ strategies.^30^ This approach promoted a highly-controlled and efficient interaction between the analytes and the sensing surface, improving sensitivity and allowing precise detection of minute concentrations.

The electrochemical-chemical catalytic (EC’) mechanism is often used for signal amplification and sensitivity enhancement.^32^ In this mechanism, there is an initial electron transfer (E) at the electrode surface. Following the electron transfer, there is a chemical reaction (C’) that regenerates the electroactive species.^32–34^ This regenerative cycle enables multiple turnovers of the same redox reaction. This will result in an amplification of the faradaic current beyond what is expected.^30^ The EC’ mechanism is particularly advantageous in trace detection. This catalytic reaction enhances sensitivity by coupling electron transfer with a rapid catalytic chemical step.

In this study, we introduce a novel approach to further lower the LOD to attomolar levels of analyte by using partitioning kinetics. We used cyclic voltammetry to perform electrochemical measurements, with a bulk phase containing trace levels of decamethyl ferrocene ((Cp*)_2_Fe^II^) alongside sodium perchlorate, to maintain electroneutrality. Given (Cp*)_2_Fe^II^'s insolubility in water, we used ICP-MS to precisely determine its unknown concentration in aqueous solution. By establishing a known concentration of (Cp*)_2_Fe^II^ in water, the concentration of (Cp*)_2_Fe^II^ was diluted down to attomolar levels by performing serial dilutions. (Cp*)_2_Fe^II^ molecules are highly soluble in an organic phase in contrast to the bulk aqueous phase. A neat 1,2-dichloroethane droplet was dispensed onto a 6.25 μm ultramicroelectrode (UME) submerged in the bulk aqueous phase containing trace amounts of (Cp*)_2_Fe^II^ molecules. Due to the intrinsically higher solubility of (Cp*)_2_Fe^II^ molecules in the organic phase, the molecules partition from the bulk aqueous phase into the oil microdroplet. Cyclic voltammetry was used to detect the presence of this analyte as the microdroplet dissolved. Voltammetry shows amplified redox peaks at attomolar concentrations because of higher concentrations in the microdroplet. This also heighten sensitivity at these low levels. To explore signal amplification, we examined the effect of oxygen, observing a marked change in peak current for the redox-active analyte with the removal and addition of O_2_. Previously, we showed detection of sub-nM levels of redox activity analyte and pushed the LOD levels to <1000 molecules of analyte in subsequent work using EC’ signal amplification strategy with ferrocyanide. In this work, we detected attomolar concentration with a significantly higher peak charge, indicating enhanced signal amplification. The amplification is attributed to oxygen which can be depicted in eqn (1) below:1
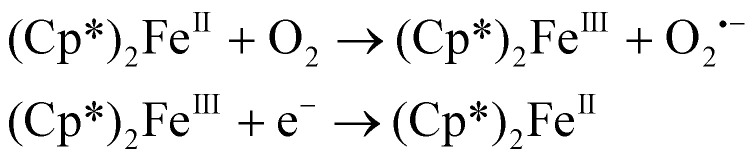


(Cp*)_2_Fe^II^ has been shown to reduce molecular oxygen to hydrogen peroxide with the reaction proceeding through a protonated intermediate ((Cp*)_2_Fe^II^ H^+^). This transformation is supported by both experimental and computational studies, which demonstrates the ability of (Cp*)_2_Fe^II^  to reduce oxygen under confined or biphasic conditions.^35^ We propose oxygen reacts with (Cp*)_2_Fe^II^ generating (Cp*)_2_Fe^III^ and O_2_˙^−^. The electron transfer at the electrode surface regenerates (Cp*)_2_Fe^II^, sustaining the redox cycle. Since the amount of O_2_ is much higher than that of (Cp*)_2_Fe^II^, the probability of the reaction occurring increases. Overall, this study introduces an analytical technique for trace compound detection, offering a meaningful contribution to advancements in electrochemical analysis.

## Materials and methods

All aqueous solutions were prepared using ultra-pure deionized water with a resistivity of 18.2 MΩ cm, sourced from a GenPure water purification system manufactured by Millipore. The organic solvent 1,2-dichloroethane, 99.8% purity (DCE) was acquired from Sigma Aldrich. The salts for all the experiments; decamethyl ferrocene ((Cp*)_2_Fe^II^), was obtained from Sigma Aldrich. Sodium Perchlorate (NaClO_4_) was obtained from Sigma Aldrich. All reagents were of analytical grade and were used without any additional purification. Prior to experimentation, the glassware underwent meticulous cleaning using mQ water, followed by acetone (99.9%, Sigma-Aldrich), and finally with the relevant solvent for each solution. Gold working electrodes with a diameter of 12.5 μm were obtained from CH Instruments, while the Ag/AgCl reference electrode in 1 M KCl, was purchased from the same supplier and was employed as the counter/reference electrode. Before usage, the working electrodes were polished with a 0.05 μm alumina powder suspension (electron microscopy sciences) on micro-cloth polishing pad (Buehler) using water. Subsequently, they underwent a cleaning process with piranha solution, which was a mixture of concentrated sulfuric acid with 30% hydrogen peroxide in a 3 : 1 ratio, to ensure thorough purification. The lab-made electrochemical cell, constructed out of Teflon, was also carefully cleaned using Piranha solution to eliminate any potential impurities. Microinjection experiments were performed using a micro-injector (FemtoJet 4i Eppendorf) and microinjection capillary tips with an orifice diameter of 10 μm (Eppendorf Femtotips). The position of the microinjector was controlled using an XYZ micro-positioning system (InjectMan 4) and monitored with an optical microscope equipped with a high-resolution sCMOS camera (C15440 Orca Fusion BT). All electrochemical experiments were conducted using a CHI 6284E potentiostat (CH Instruments). The reference electrode was placed in a separate compartment containing 1 M KCl and was connected to the cell through a salt bridge. The salt bridge was created by filling a glass tube with 3% agarose (99.9%, Sigma-Aldrich) containing 1 M potassium chloride. Inductively coupled plasma-mass spectra were obtained using a Thermo Sceintific Element II Inductively Coupled Plasma-Mass Spectrometer (Research Instrumentation Center, Purdue University, IN) and all samples were analyzed in 2% HNO_3_ using iron as an internal standard.

## Results and discussion

**Fig. 1:**
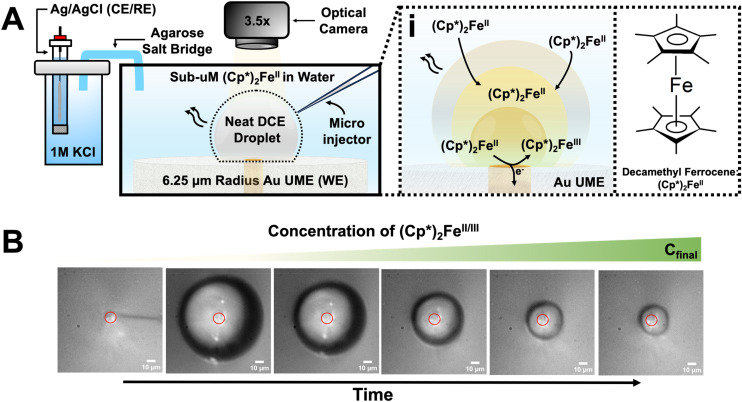
(A) The cell contains an Au UME as the WE, microinjector, and an agarose salt bridge connecting to a 1 M KCl reservoir with the CE/RE, Ag/AgCl. Experiment monitoring is facilitated by an optical camera. The reagents of the experiment comprise of (Cp*)_2_Fe^II^ in the aqueous bulk phase and a neat DCE droplet. (i) Chemical structure of (Cp*)_2_Fe^II^ is shown and partitioning of (Cp*)_2_Fe^II^ into the DCE droplet is illustrated. (B) Micrographs for different droplet sizes are shown along with time showing that the droplet becomes smaller over time and concentration increases over time.

The experimental setup is shown in Fig. 1(A), where the bulk phase consists of trace (Cp*)_2_Fe^II^ along with 10 mM NaClO_4_. A neat DCE droplet is injected and positioned onto a 6.25 μm radius Au UME using a microinjector, as shown in Fig. 1(A). While the DCE droplet spontaneously dissolves into the bulk aqueous phase, (Cp*)_2_Fe^II^ continuously partitions into the DCE droplet. The partitioning of (Cp*)_2_Fe(ii) into the DCE droplet was observed to occur almost instantaneously. It is remarkable how rapidly (Cp*)_2_Fe(ii) migrates to and partitions into the DCE droplet. The moment a DCE droplet was introduced onto the electrode surface, a clear electrochemical response corresponding to the oxidation of (Cp*)_2_Fe(ii) to (Cp*)_2_Fe(iii) was immediately detected, indicating swift phase transfer and redox activity at the interface. The CV for (Cp*)_2_Fe^II^ in bulk is shown in Fig. S1. The dissolution of DCE in water is driven by its solubility limit (0.869 g per 100 mL at 20 °C). During droplet dissolution, electrochemical measurements are conducted using a two-electrode setup, with the Au UME as the working electrode and Ag/AgCl in 1 M KCl, connected by an agarose salt bridge, as the reference/counter electrode. The reference electrode was placed in a separate reservoir connected by a salt bridge as a precautionary measure to minimize any potential impurities affecting our electrochemical signal. While placing the reference electrode directly in the main cell is also acceptable, the use of a separate reservoir was chosen to reduce the risk of potential impurities. Real-time monitoring of the droplet's geometry is captured by a high-resolution camera, which acquires bright-field micrographs using diffused white light illumination. The bulk aqueous solution is prepared by adding a small amount of (Cp*)_2_Fe^II^ to water, followed by stirring for 30 minutes to ensure trace amounts of (Cp*)_2_Fe^II^ dissolve in water. While (Cp*)_2_Fe^II^ is generally insoluble in water, trace amounts will still dissolve in it, as shown later. Upon pipetting a DCE droplet onto the Au UME, (Cp*)_2_Fe^II^ surrounding the droplet immediately partitions into the DCE droplet, as DCE is a more favorable solvent than water, as shown in inset (i) in Fig. 1(A).

During the dissolution of the DCE microdroplet, cyclic voltammograms are acquired around the formal potential of redox couple (Cp*)_2_Fe^III^/(Cp*)_2_Fe^II^. During the oxidation of (Cp*)_2_Fe^II^ to (Cp*)_2_Fe^III^, ClO_4_^−^ ions partition from the aqueous phase into the oil phase to maintain electroneutrality with (Cp*)_2_Fe^III^, and *vice versa*. As the droplet dissolves, (Cp*)_2_Fe^II^ continues to partition into the DCE droplet until it fully dissolves. During this process, the concentration of the redox active analyte is enriched in the microdroplet. An example of this process is shown in Fig. 1(B), which shows the micrographs of the droplet at different time points, showing varying droplet sizes. As the droplet decreases in size, concentration enrichment of (Cp*)_2_Fe^II/III^ occurs due to partitioning and droplet dissolution, as qualitatively illustrated by the color gradient marking the total concentration of (Cp*)_2_Fe^II/III^ molecules in the microdroplet.

**Fig. 2:**
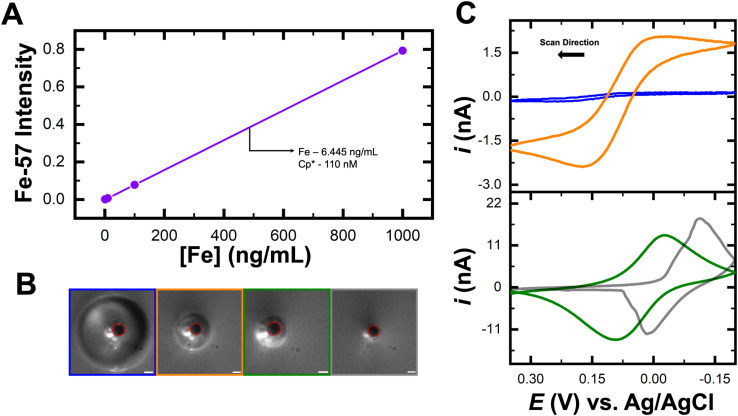
(A) ICP-MS calibration curve for the determination of trace Fe in unknown solution. (B) Optical micrographs recorded for the dissolution of a neat DCE droplet as (Cp*)_2_Fe^II^ continuously partition into the droplet from the bulk phase. The scale bar for the micrographs is 10 μm (C) Cyclic voltammograms were recorded at different time points over an 11 minute dissolution period to show electrochemical response at starting radius of 51 μm droplet size to near complete dissolution of the droplet at 7 μm radius droplet size.

A challenge in studying trace detection for this system is that the concentration of (Cp*)_2_Fe^II^ dissolved in water is unknown, as (Cp*)_2_Fe^II^ is insoluble in water. To determine the concentration of dissolved (Cp*)_2_Fe^II^, we used ICP-MS to measure the Fe concentration. A calibration curve was developed to correlate signal intensity with Fe analyte concentration. The unknown sample, containing a trace amount of (Cp*)_2_Fe^II^ in water, was analyzed by ICP-MS, yielding an Fe concentration of 6.445 ng mL^−1^. Using the calibration curve shown in Fig. 2(A), the concentration of (Cp*)_2_Fe^II^ was calculated to be 110 nM. The details for the calibration curve can be shown in Table S1. A bulk measurement was taken of 110 nM (Cp*)_2_Fe^II^ to show it cannot be detected bulk under standard conditions or voltammetry of 1 mM (Cp*)_2_Fe^II^ solution where a sigmoid is present showcasing oxidation of (Cp*)_2_Fe^II^, which is shown in Fig. S2. An experiment was then conducted to observe the partitioning of 110 nM (Cp*)_2_Fe^II^ into the DCE droplet. The setup included an aqueous bulk phase containing 10 mM NaClO_4_ in water and 110 nM (Cp*)_2_Fe^II^, with a DCE droplet positioned on the UME. The apparent standard potential for the redox couple (Cp*)_2_Fe^III^/(Cp*)_2_Fe^II^ is observed at −0.1 V *vs.* Ag/AgCl.^27^ A DCE droplet was injected onto the electrode surface, where it spontaneously dissolved over time, allowing (Cp*)_2_Fe^II^ to partition into it. Optical micrographs acquired during droplet dissolution are shown in Fig. 2(B), with the electrode position marked by a solid red circle at the center of each micrograph. The initial droplet size was measured at 85 μm based on the blue micrograph in panel (B) of Fig. 2. The UME potential was continuously scanned between −0.2 V and 0.35 V *vs.* Ag/AgCl at a rate of 0.2 V s^−1^ to monitor the effect of droplet dissolution on the electrochemical response of the redox couple confined in the droplet. A total of 125 voltammograms were recorded; out of these, four representative curves are presented in Fig. 2(C). The blue and orange curves correspond to CV36 and CV101, respectively, while the green and gray curves represent CV117 and CV120.

When a neat DCE droplet was injected onto the electrode, rapid partitioning of (Cp*)_2_Fe^II^ occurred, indicated by the sigmoid formation in CV36 (blue voltammogram). As the droplet continued to dissolve, concentration enrichment occurred alongside ongoing partitioning of (Cp*)_2_Fe^II^ into the DCE droplet. This effect is evident in CV101 (orange voltammogram), where the sigmoidal peaks begin to broaden. The voltammograms then transitioned into a duck-shaped curve, shown in CV117 (green voltammogram). The final transition from a duck-shaped curve to Gaussian-shaped peaks, as seen in CV120 (gray voltammogram), suggests thin-layer behavior. Once thin-layer behavior is observed, the droplet completely dissolves, and no redox activity is detected. In essence, as the droplet dissolves, partitioning of (Cp*)_2_Fe^II^ into the DCE phase continues, resulting in an enriched concentration within the droplet. An additional experiment was performed to show 110 nM concentration of (Cp*)_2_Fe^II^ can be detected in a dissolving droplet, shown in Fig. S3. There is an initial nonzero current that was observed in the voltammograms. This is attributed to capacitive charging at the surface of the UME. Furthermore, the presence of a potential gradient across the DCE–water interface promotes ion transfer. The ClO_4_^−^ ion transfer influences the electrochemical environment within the droplet. As (Cp*)_2_Fe^II^ accumulates in the droplet over time, the local concentration increases.^27^ This leads to a gradual positive shift in the half-wave potential. This dynamic enrichment and interfacial ion exchange account for both the capacitive current at the start of the scan and the observed shift in 
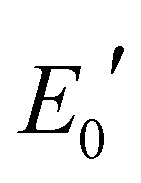
.

**Fig. 3:**
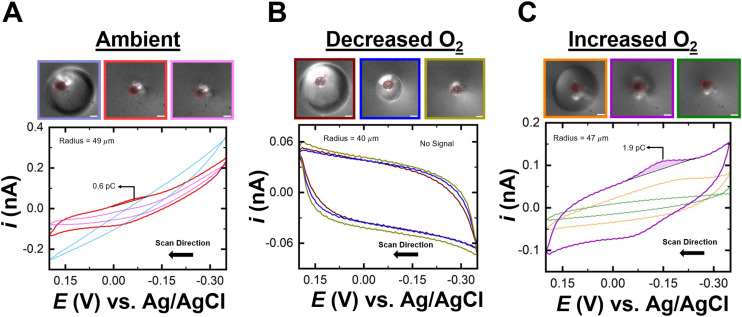
(A) Cyclic voltammograms recorded at ambient conditions with optical micrographs depicting decreasing droplet size. (B) Cyclic voltammograms recorded in argon rich conditions with optical micrographs depicting decreasing droplet size. (C) Cyclic voltammograms recorded at oxygen rich conditions. The aqueous bulk phase contains 1 aM (Cp*)_2_Fe^II^ and 10 mM NaClO_4_ in water. The scale bar for the micrographs is 10 μm.

Based on this proof of concept, the next set of experiments aimed to test ultra-low detection of (Cp*)_2_Fe^II^ by leveraging partitioning between the phases. The trace concentration of (Cp*)_2_Fe^II^ in water, determined to be 110 nM by ICP-MS, was serially diluted to a final concentration of 1 aM. Attomolar concentration represents an ultra-dilute regime in which any impurities can have detrimental effects towards analyte detection. To ensure reliable and reproducible electrochemical measurements, strict precautions were taken to minimize electrolyte impurities. All solutions were prepared using freshly obtained Milli-Q deionized water. Any transferring of liquids were handled exclusively with clean pipette tips. All droplet experiments were done with fresh DCE. A new rubber stopper was used for each experiment to prevent cross-contamination. A single working electrode was employed throughout the study and was carefully polished and cleaned between runs to maintain surface consistency. There can be minor variations observed in the initial cyclic voltammograms could be attributed to slight differences in electrode surface condition from polishing. The rigorous steps were taken to reduce contamination and minimize impurities affecting our electrochemical response. Similar to the previous experiment, a 1 aM solution of (Cp*)_2_Fe^II^ in 10 mM NaClO_4_ was used as the bulk phase. A DCE droplet was injected onto the electrode, and the potential was swept from −0.2 V to 0.35 V *vs.* Ag/AgCl at a scan rate of 1 V s^−1^. As the droplet dissolved, no electrochemical response from (Cp*)_2_Fe^II^ was observed until the droplet dissolved to a small volume, as shown in Fig. 3(A). The light blue voltammogram (CV101) and micrograph show the initial droplet size of 98 μm, yielding no redox activity. As the droplet became extremely small, approximately the size of the electrode, redox activity emerged, shown in the red voltammogram (CV238) and corresponding micrograph. The pink voltammogram (CV251) indicated a return to zero redox activity from (Cp*)_2_Fe^II^ as the droplet fully dissolved (pink micrograph). The oxidation peak measured was around 0.6 pC, higher than expected given the concentration of 1 aM and a droplet radius of 49 μm. The anticipated charge for a 1 aM solution contained within a 3 mL vessel is calculated to be 0.29 fC (see SI for calculations). These results are shown in Fig. S4. Based on our previous works, it is known that under ambient conditions (Cp*)_2_Fe^II/III^ molecules are notoriously reactive to dissolved oxygen in the water phase. This led us to conclude that a much higher charge than expected was observed due to signal amplification arising from the interaction between oxygen and (Cp*)_2_Fe^II/III^ molecules. This led us to further test the sensitivity of detection by comparing the redox activity at ambient, decreased oxygen and increased oxygen levels.

The next experiment removed O_2_ from the system to assess its influence on the signal arising from the redox activity of (Cp*)_2_Fe^II/III^ molecules. The solution of 1 aM (Cp*)_2_Fe^II^ in water with 10 mM NaClO_4_ was bubbled with argon for 20 minutes to ensure saturation with Ar. This solution was then used as the bulk phase, and a DCE droplet was placed on the electrode. As the droplet dissolved, no redox activity was observed from start to complete dissolution, as shown in Fig. 3(B). This observation was verified over tens of runs and additional voltammograms for independent experiments are presented in the SI. Additional runs are shown in Fig. S5. The brown voltammogram (CV101) and micrograph show the initial droplet with no redox activity (brown micrograph). As the droplet continued to dissolve (blue micrograph), no discernible electrochemical response from (Cp*)_2_Fe^II^ was observed, as shown in the blue voltammogram (CV251). Even when the droplet reached the size of the electrode, no redox activity was detected, as shown in the dark yellow voltammogram (CV351) and micrograph. This indicates that O_2_ likely amplifies the signal, as its removal prevented the 1 aM concentration from producing an electrochemical response. While removing O_2_ offered insights into its amplifying effect, further experiments are needed to draw a definitive conclusion.

An increase in O_2_ concentration in the solution was performed to examine the effect of additional O_2_ species on signal amplification. The solution of 1 aM (Cp*)_2_Fe^II^ in water with 10 mM NaClO_4_ was bubbled with O_2_ gas for 20 minutes. Once saturated with O_2_, the solution was added to the cell, and a neat DCE droplet was placed on the electrode. Initially, the droplet, with a size of 94 μm, showed no redox activity, as shown in the orange voltammogram and micrograph in Fig. 3(C). When the droplet further dissolved, a clear oxidation and reduction peak appeared, shown in the purple voltammogram (CV255) and corresponding micrograph. The green voltammogram (CV259) indicates the point at which the droplet had fully dissolved, causing the electrochemical response to return to the bulk solution, as shown in the green micrograph. The oxidation peak charge for the purple voltammogram was recorded at 1.9 picocoulombs, approximately three times the signal observed at ambient O_2_ levels. The droplet sizes were found to be comparable under both conditions: ambient and increased oxygen conditions. At ambient conditions, the droplets had a radius of 49 μm, while at increased oxygen conditions, the radius was slightly smaller but similar at 47 μm. Due to their similar sizes, both droplets fully dissolved within 11 minutes. The recorded charge at ambient conditions was 0.6 pC, whereas at increased oxygen conditions, it was 1.9 pC, a threefold increase compared to ambient conditions. This result demonstrated that O_2_ saturation significantly amplified the electrochemical signal. When the aqueous phase was saturated with O_2_ to investigate its influence on the detection of 1 aM (Cp*)_2_Fe^II^. The presence of dissolved oxygen significantly impacted the kinetics of the EC’ reaction, particularly the chemical (C’) step that follows the initial electron transfer. Oxygen will act as a competing reactant or quencher, which will control the rate of the subsequent chemical transformation. This effect on the reaction kinetics is shown in the rate expression (eqn. (2)) below:

2 Rate = *k* [(Cp*)_2_Fe^ii^][O_2_]

As the chemical step is accelerated by the higher availability of oxygen, the system will produce an increase in catalytic current. This rise in current reflects the enhanced turnover of the redox-active species, further confirming that the EC’ process is chemically and kinetically controlled under these conditions. Based on these results, it is evident that O_2_ facilitates signal amplification across several orders of magnitude, suggesting that the true LOD for this methodology is likely lower than attomolar levels. The influence of dissolved oxygen on the electrochemical signal is strongly affected by the concentration of the redox-active species. At lower concentrations of (Cp*)_2_Fe^II^, the contribution of oxygen reduction becomes more prominent. The faradaic current of the redox-active species is smaller and more susceptible to interference. In contrast, at higher (Cp*)_2_Fe^II^ concentrations, the electrochemical response is strongly influenced by the oxidation of (Cp*)_2_Fe^II^. This will suppress oxygen's influence on electrochemical response. This is due to the faradaic current associated with (Cp*)_2_Fe^II^ which will have a more prominent effect than that from oxygen reduction reaction. As such, oxygen has a less effect on the voltammetric behavior at high (Cp*)_2_Fe^II^ levels. Instead, oxygen will have a larger impact at dilute conditions. Furthermore, the precise mechanism underlying signal amplification in the presence of O_2_ remains under investigation and will be explored in future studies from our group.

## Conclusion

In summary, this study demonstrates a novel analytical strategy leveraging partitioning and EC’ catalytic mechanism to achieve ultra-sensitive detection of a redox analyte at 1 aM concentration through electrochemical analysis. By enabling the partitioning of (Cp*)_2_Fe^II^ from the bulk phase into a neat 1,2-dichloroethane microdroplet on a gold microelectrode, coupled with an EC’ mechanism with O_2_, exceptional sensitivity was attained. The amplification of the electrochemical signal was further enhanced by saturating the aqueous phase with O_2_, underscoring the synergistic role of O_2_-driven signal amplification and concentration enrichment *via* dissolving droplets. These findings highlight a powerful approach for achieving extraordinarily low detection limits, paving the way for advanced applications in sensor technologies, such as medical diagnostics and environmental monitoring.

## Author contributions

All authors have agreed to the final version of the manuscript.

## Conflicts of interest

There are no conflicts to declare.

## Note added after first publication

This article replaces the version published on 1st September 2025 to correct the presentation of eqn (1). This does not impact the contents or conclusions in any way.

## Supplementary Material

AN-150-D5AN00795J-s001

## Data Availability

The authors confirm that the data supporting the findings of this study are available within the article and its SI. The SI contains cyclic voltammograms, ICP-MS tabulated values, and calculations. See DOI: https://doi.org/10.1039/d5an00795j. Raw data that support the findings of this study are available from the corresponding author, upon reasonable request.
