# Supplementary material for: Dissolving microdroplet electroanalysis enables attomolar-level detection
Source: Analyst. 2025 Aug 19;150(19):4285–92. doi: 10.1039/d5an00795j (PMC12401217; doi:10.1039/d5an00795j)
Supplement: AN-150-D5AN00795J-s001 [file AN-150-D5AN00795J-s001.pdf]

Supplementary Information (SI) for Analyst.
This journal is © The Royal Society of Chemistry 2025

Supplementary Information

Dissolving Microdroplet Electroanalysis Enables Attomolar-Level Detection

James H. Nguyen[a,^], Ashutosh Rana[a,^], Savannah M. Hatch[a] and Jeffrey E. Dick[a,b*]

[a]Department of Chemistry, Purdue University, West Lafayette, IN, 47907, USA
[b]Elmore Family School of Electrical and Computer Engineering, Purdue University, West Lafayette, IN, 47907, USA
*Corresponding Author(s)
Jeffrey E. Dick (jdick@purdue.edu)

Table of Contents

Figure or Theory: Description	Page Number
**Figure S1:** Cyclic voltammogram of 1mM $(Cp^*)_2Fe^{(II)}$ in DCE bulk	S2
**Table S1:** ICP-MS tabulated values of Fe-57 and [Fe] (ng/mL) for unknown concentration of $(Cp^*)_2Fe^{(II)}$ in aqueous bulk	S3
**Figure S2:** Cyclic voltammogram of 110 nM $(Cp^*)_2Fe^{(II)}$ in an aqueous bulk with 10 mM $NaClO_4$.	S4
**Figure S3:** Cyclic voltammogram of 110 nM $(Cp^*)_2Fe^{(II)}$ in an aqueous bulk with 10 mM $NaClO_4$ when a neat DCE droplet was added.	S5
**Figure S4:** Calculations for anticipated charge for 1 aM solution within a 3 mL vessel	S6
**Figure S5:** Cyclic voltammograms of 1 aM $(Cp^*)_2Fe^{(II)}$ in DCE droplet in an aqueous bulk with 10 mM $NaClO_4$ at ambient, decreased, and increased O_2	S7

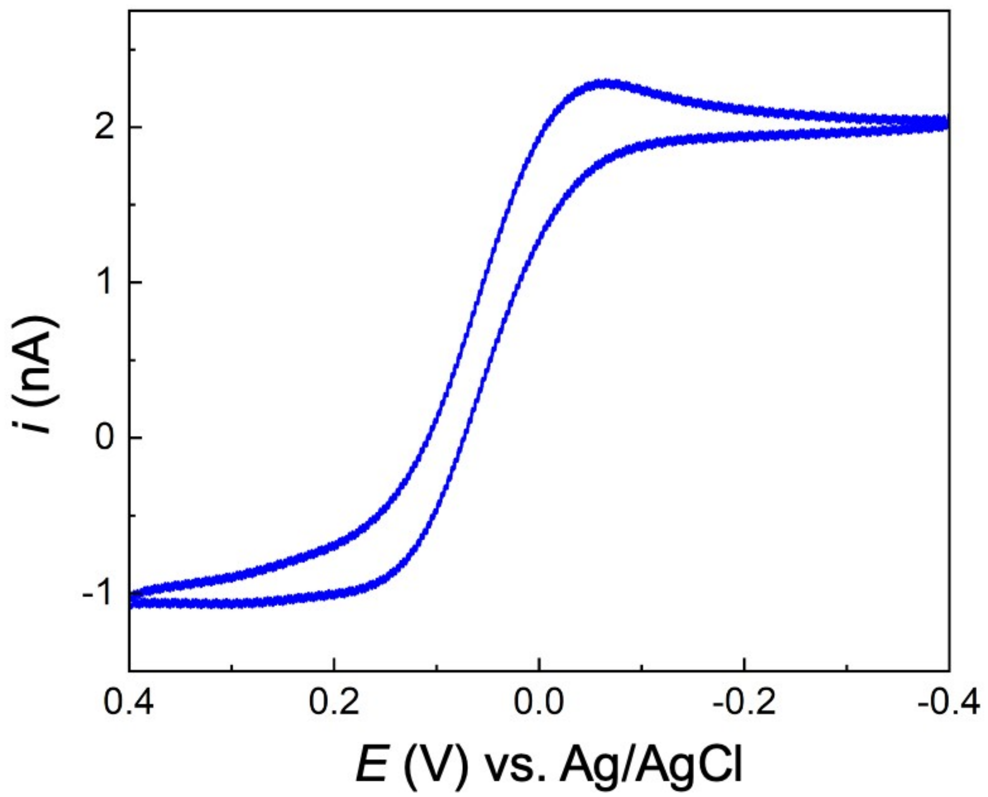

Figure S1. Cyclic voltammogram recorded of 1 mM $(Cp^*)_2Fe^{II}$ in DCE bulk phase

Table S1. ICP-MS tabulated values of Fe-57 and [Fe] (ng/mL) for unknown concentrations of $(Cp^*)_2Fe^{II}$ in aqueous bulk for the calibration curve

Standard Fe Concentration (ppb, ng/mL)	Fe57 Intensity, internal control normalized/method blank corrected
0	0.0000
1	0.0001
10	0.0070
100	0.0775
1000	0.7930

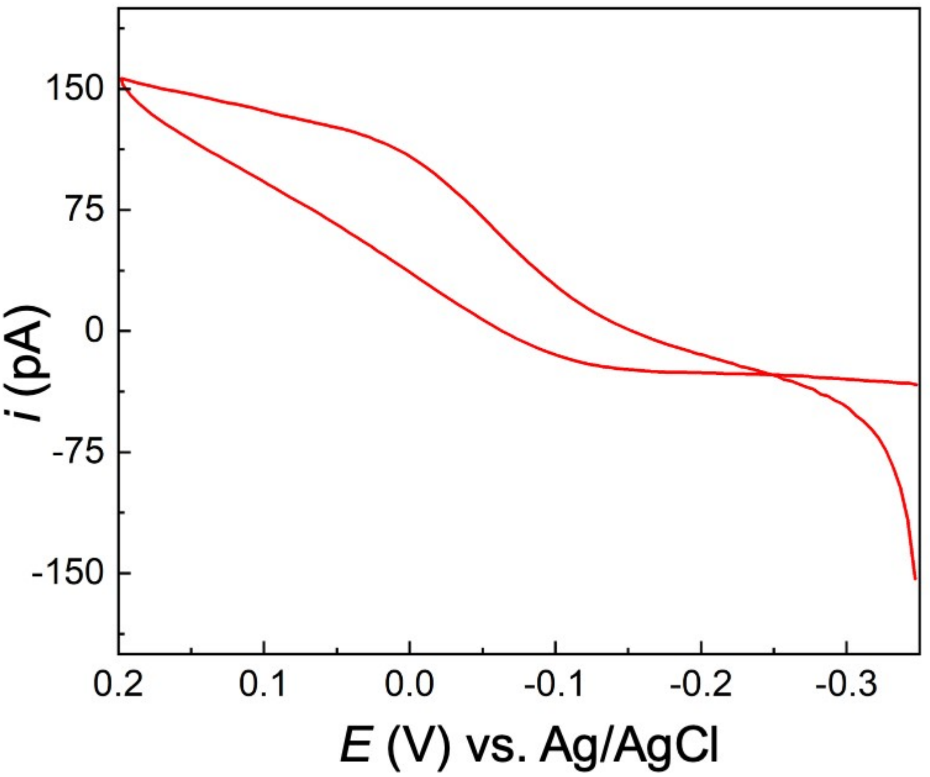

Figure S2. Cyclic voltammogram recorded at 110 nM $(Cp^{*})_2Fe^{II}$ in aqueous bulk

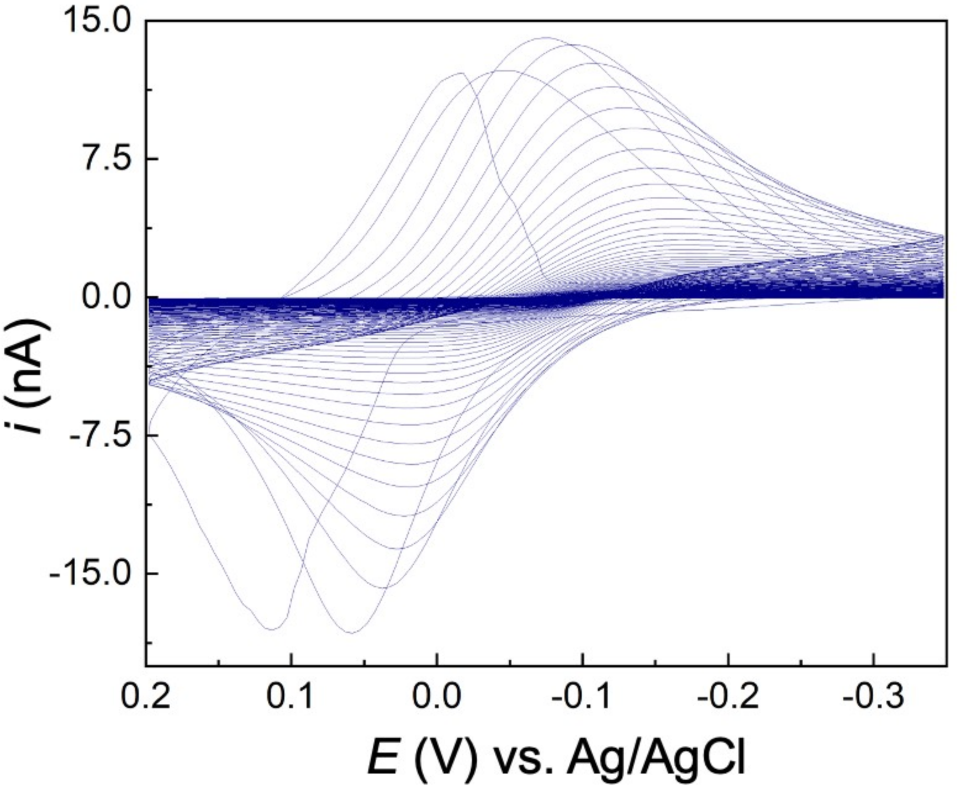

Figure S3. Cyclic voltammograms of 110 nM $(Cp^*)_2Fe^{II}$ in an aqueous bulk with 10 mM $NaClO_4$ when a neat DCE droplet was injected on the electrode

$$Q = nF \tag{1}$$

$$Q = [Concentration] * volume * F \quad (2)$$

$$Q = 10^{18}\left(\frac{mol}{L}\right) * 0.003\ L * 96485\ (\frac{C}{mol}) \quad (3)$$

$$Q = 2.9 * 10^{-16}\ or\ 0.29\ fC \quad (4)$$

Figure S4. Calculations for the anticipated charge of a 1 aM solution within a 3 mL vessel

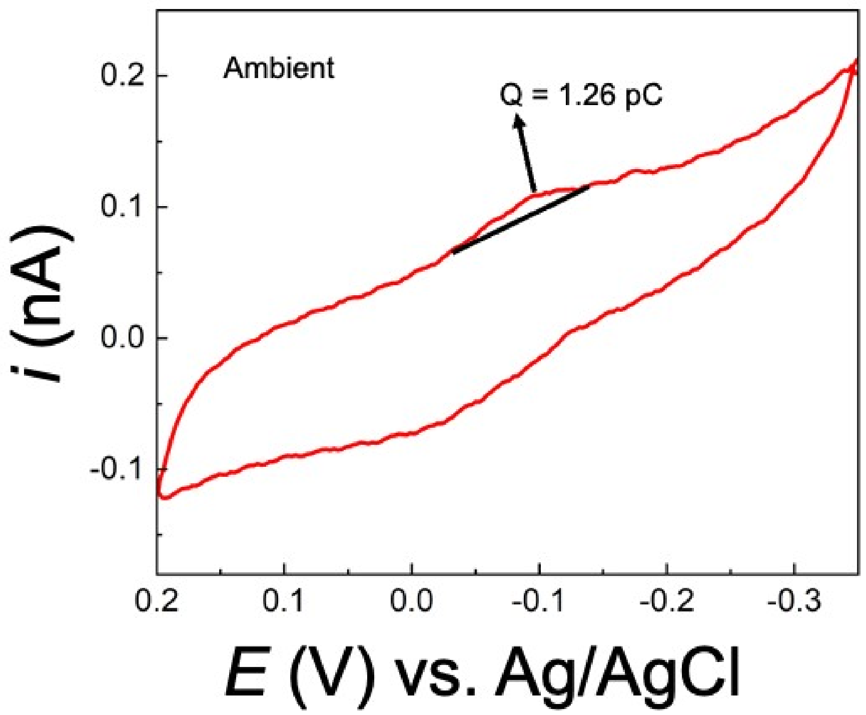
Ambient
Q = 1.26 pC
0.2
0.1
0.0
-0.1
i (nA)
0.2
0.1
0.0
-0.1
-0.2
-0.3
E (V) vs. Ag/AgCl

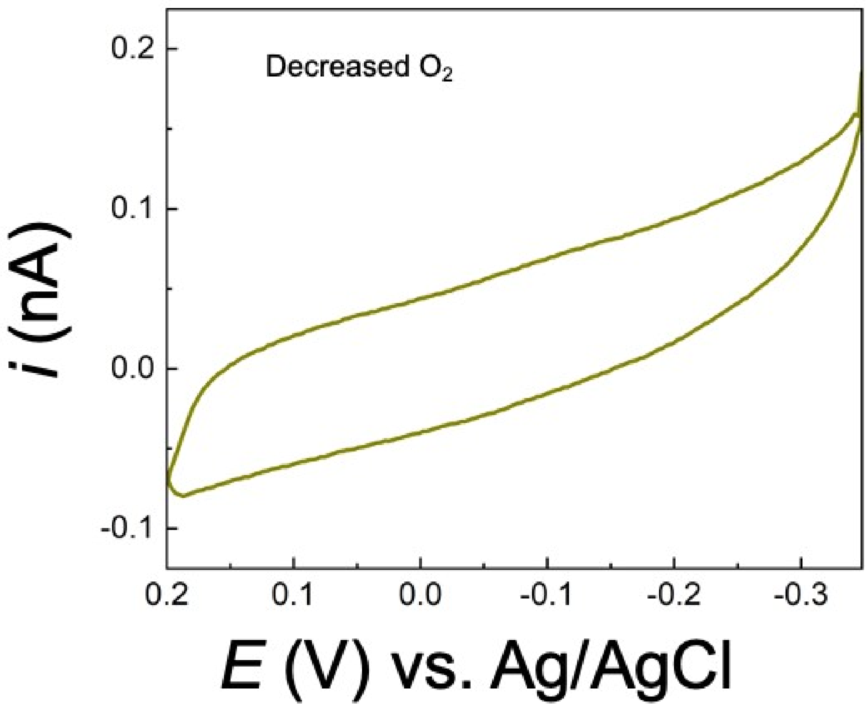
Decreased O2
0.2
0.1
0.0
-0.1
i (nA)
0.2
0.1
0.0
-0.1
-0.2
-0.3
E (V) vs. Ag/AgCl

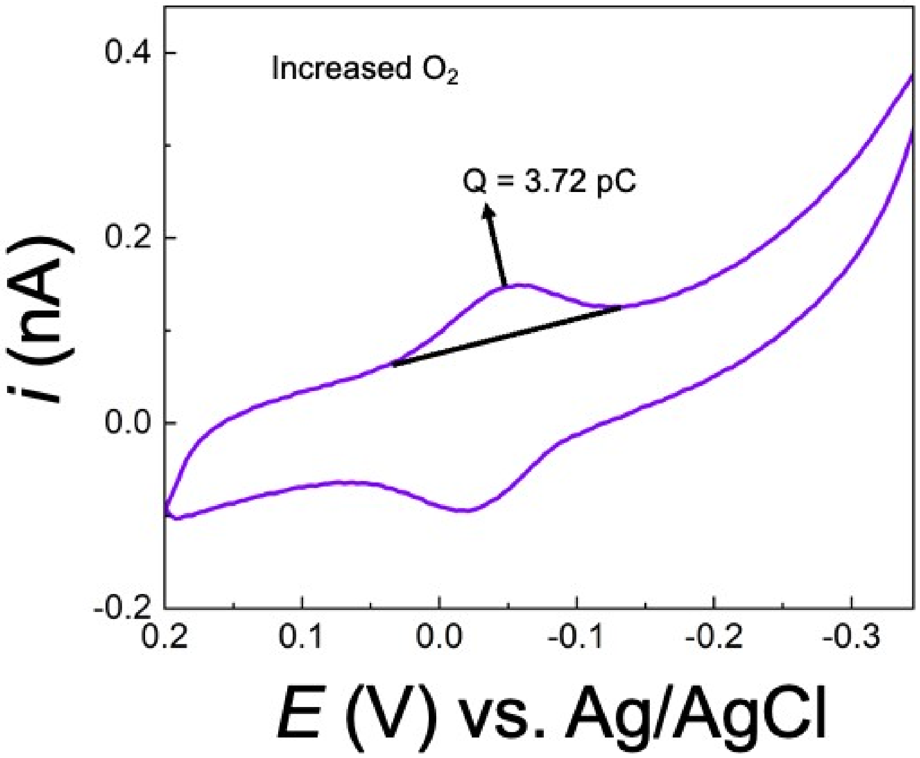

Figure S5: Cyclic voltammograms of 1 aM $(Cp^*)_2Fe^{(II)}$ in DCE droplet in an aqueous bulk with 10 mM $NaClO_4$ at ambient, decreased, and increased O_2. The peak current can be shown for both ambient and increased O_2 which shows a higher charge for when the system is saturated with O_2.
